# Prevalence and correlates of maternal early stimulation behaviors during pregnancy in northern Ghana: a cross-sectional survey

**DOI:** 10.1186/s12884-020-03476-9

**Published:** 2021-01-04

**Authors:** Jessica Mackness, John A. Gallis, Raymond Kofi Owusu, Mohammed Ali, Safiyatu Abubakr-Bibilazu, Haliq Adam, Raymond Aborigo, John Koku Awoonor-Williams, Margaret Lillie, Elena McEwan, John Hembling, Lavanya Vasudevan, Joy Noel Baumgartner

**Affiliations:** 1 Duke Global Health Institute, Duke University, Durham, NC USA; 2 Department of Biostatistics & Bioinformatics, Duke University, Durham, NC USA; 3 Catholic Relief Services Country Office, Tamale, Ghana; 4 Navrongo Health Research Centre, Navrongo, Ghana; 5 Ghana Health Service, Accra, Ghana; 6 Catholic Relief Services Head Quarters, Baltimore, MD USA; 7 Department of Family Medicine & Community Health, Duke University, Durham, North Carolina USA

**Keywords:** Early stimulation behaviors, Early childhood development, Maternal-fetal attachment, Intimate partner violence, Pregnancy

## Abstract

**Background:**

Per UNICEF’s Nurturing Care Framework, early childhood development (ECD) begins during pregnancy and many lower-resource settings need data to inform their programs for optimal child development. The maternal-fetal relationship can be partly examined via a series of bonding activities called early stimulation behaviors (ESB). This study describes early stimulation behaviors and the associated correlates among pregnant women in Ghana.

**Methods:**

This cross-sectional study used data from a cluster-randomized trial in two districts of Northern Ghana. A total of 374 pregnant women were enrolled at baseline and administered a pre-intervention survey. Communication-related early stimulation behaviors was the primary outcome which was evaluated using three maternal-fetal bonding activities; did the woman self-report touching and/or talking, singing, and/or talking about family to her belly. A generalized estimating equation modified Poisson model was used for the bivariate and multivariable analysis.

**Results:**

About half of the participants reported performing communication-related ESB during pregnancy frequently or sometimes. Bivariate analysis revealed that negative life experiences including higher rates of emotional, physical and sexual intimate partner violence (IPV) and having moderate to severe depressive symptoms were associated with women performing early stimulation behaviors more often. In the multivariable model, physical intimate partner violence remained significantly associated with early stimulation behaviors.

**Conclusion:**

Research on early stimulation behaviors is still in a nascent phase. It is unclear why our results revealed an association between intimate partner violence and early stimulation behaviors; this could reflect a coping mechanism for the expectant mother. Further research is needed to better understand this association and explore potential long-term impacts of early stimulation behaviors during pregnancy on child development.

**Trial registration:**

Clinical Trials # NCT03665246, August 29, 2018.

**Supplementary Information:**

The online version contains supplementary material available at 10.1186/s12884-020-03476-9.

## Background

Early childhood development (ECD) is defined by the Nurturing Care Framework as ‘children’s cognitive, physical, language, motor, and social and emotional development', between conception and age 8 [1]. Despite major contributions in ECD in recent years, there is a gap in knowledge around caregiver characteristics from conception to birth that contribute to optimal development. One of the core domains of the Nurturing Care Framework is responsive caregiving, which includes caregiver sensitivity and stimulating activities that encourage play and communication. The maternal-fetal relationship, which focuses on interactions and feelings the expectant mother has towards her developing fetus, is one the first opportunities in ECD to create a nurturing relationship between caregiver and baby [2]. This relationship also opens a window for pregnant women to begin performing early stimulation behaviors (ESB), like touching and talking to her belly during pregnancy. Currently most ECD programs begin at birth; however, evidence suggests that for nurturing care to be most effective, interventions need to begin during pregnancy and be caregiver-centered [3].

The World Health Organization (WHO) recently published guidelines on how to strengthen policies and programs to achieve better developmental outcomes for all children [4]. Focusing on ECD creates a foundation for health, well-being, and productivity from childhood to adulthood [1]. Investing in ECD is the most efficient way to create the human capital needed to grow economies and eliminate extreme poverty and inequities in societies [5, 6].

Per the Nurturing Care Framework, ECD begins at conception [1]. The prenatal period is a time of rapid brain development that leads to neural processing capabilities and the ability to learn [7]. Touch and sound are some of the first senses to develop in the womb and studies have shown that when a fetus is exposed to stimuli like voices at 34 weeks gestational age, the newborn will subsequently perceive the stimulus similarly to that experienced prenatally [7, 8]. This demonstrates that learning and cognitive functions like short and long-term memory begin during pregnancy. A systematic review of maternal-fetal attachment (MFA) and infant developmental outcomes suggested that there is some evidence that higher maternal and fetal bonding during pregnancy is related to higher developmental outcomes for the subsequently born child; however, there was a call for more methodologically sound research to strengthen the evidence base [9]. Beyond the potential early attachment issues during pregnancy, early stimulation behaviors during pregnancy may help lay the ‘groundwork’ for responsive caregiving practices during infancy when stimulating activities are even more crucial for early childhood development.

Evidence indicates that maternal mood during pregnancy can have negative impacts on the maternal-fetal relationship and consequently, lead to lower rates of bonding postnatally [10, 11]. However, there are several ways for pregnant women to enhance their mood and bond with their fetus during pregnancy; simple activities like touching and talking to her belly or any activity she finds joyous. Performing joyous or ‘pleasant activities’ is a common cognitive behavioral therapy approach that has been shown to lower perinatal depression [12]. Prenatal bonding activities enhance maternal-fetal attachment, especially at higher gestational ages when the expectant mother can feel the movement of their child [13, 14]. Early stimulation behaviors during pregnancy and maternal-fetal attachment are widely understudied and more research is needed to better understand associated factors and long-term impact on developmental outcomes for children, particularly in low-resource settings.

Currently, 23% of young children are at risk of not meeting their full developmental potential in Ghana [13, 14]. Data on responsive caregiving within the Nurturing Care Framework in Ghana is limited [15]. The objectives of this paper are to describe early stimulation behaviors (ESB) during pregnancy among women of Nabdam and West Mamprusi Districts of Northern Ghana and identify associated correlates of ESB. Ideally, these findings will inform future ECD programs that may begin during pregnancy and contribute to helping children reach their full developmental potential.

## Methods

### Study overview

This study uses cross-sectional pre-intervention baseline data from a parent study that is a longitudinal, cluster randomized control trial (Clinical Trials # NCT03665246) to evaluate the impact of a maternal mental health/ECD intervention called Integrated Mothers and Babies Course (iMBC) [16, 17]. The parent trial included 32 communities/clusters with 16 clusters per arm. The average cluster size was over 11 per group, leading to a final baseline sample size of 374 and > 90% power for the parent study’s primary outcome (maternal mental health) [18].

The study was a collaboration between Duke University and Catholic Relief Services (CRS). CRS-Ghana implemented the intervention with approval and support from the Ghana Health Service. IMBC is part of CRS’ Rural Emergency Health Service and Transport (REST II) project that aims to scale-up community-based approaches to health services for improved maternal, newborn, child health, and nutrition practices. A priority activity of REST II is Community Surveillance and Targeted Education Sessions (C-PrES) in each community. The parent study randomized clusters such that women in the iMBC groups received all the same information as the standard C-PrES groups, but with the added iMBC intervention, which had content on stressors during pregnancy and how to better manage those stressors to decrease the risk of future depression. This manuscript adheres to the STROBE statement guidelines for reporting cross-sectional studies [19].

### Study setting & participants

Pre-intervention baseline data was collected between August 29 and September 10, 2018 among 32 communities in the West Mamprusi Municipality and the Nabdam District of Northern Ghana. These areas are considered rural populations where a majority of the residents are in the lowest wealth quantile and have high infant mortality rates [20]. Participant inclusion criteria included being pregnant at baseline, 16 years or older, planned to attend C-PrES groups at the time of the baseline survey, and planned to maintain residence in the community for at least 6 months.

### Procedures

Participants gave written informed consent via signature or thumbprint with a witness. Interviewer-administered surveys were conducted by research assistants (RAs) in the local languages (Mampruli and Nabt) on Samsung Galaxy tablets. The Mampruli and Nabt languages are not commonly written, therefore, the survey was written in English and was vocally translated at the time of each interview by the local research assistants. Before data collection began, all 24 RAs participated in a week-long training in which they reviewed the survey content, practiced administering the survey in English and, and then practiced administering the survey in their local language. In addition, mid-week during the training, all research assistants participated in pre-testing of the survey in the community in order to finalize any edits on the survey and to ensure that all research assistants were familiar with and could debrief about their common understanding of the survey items prior to formal data collection. Please see the Supplementary Materials for the full survey. Data was captured by using CommCare, a HIPAA (The United States Health Insurance Portability and Accountability Act of 1996) compliant, cloud-based, and field data collection and reporting platform, published by Dimagi, Inc. Surveys were conducted at participants’ homes or at another agreed upon private location to ensure confidentiality and participants were compensated with two bars of soap.

Study risks were minimal; however, for the mental health assessment, if participants endorsed suicidal ideation, RAs provided a referral to district social welfare and mental health officers for further support per guidelines from the Ghana Health Service who attended the RA training. Similarly, for domestic violence survey items, if participants indicated physical or sexual violence, they were offered a referral to the Domestic Violence and Victim Support Unit through the District Gender Officer, which aligned with Ghana Health Service recommendations and global best practices for gender-based violence research [21].

### Ethical approval

Ethical approvals were received from the Duke University Campus IRB (ID# 2019–0020) and the Navrongo Health Research Centre Institutional Review Board (ID # NHRCIRB314).

### Measures

The main outcome variable was early stimulation behaviors performed by the pregnant women towards their pregnant belly. Throughout this paper, we will use the term ‘pregnant belly’ because that is how it is referred to programmatically and in the questionnaire. These behaviors included touching/talking to her belly, singing songs, dancing, and talking to her belly about family. These were example ESB that Catholic Relief Services used in the iMBC sessions and have used in other programmatic materials [17]. As part of the original survey, two additional questions were asked regarding whether the father touches/talks to the expectant mother’s belly and if other children touch/talk to the belly (part of CRS program materials). It was decided not to include these two questions beyond descriptive analysis due to confounding factors such as living situation, parity and relationship status that could interfere with the results. Women were asked to self-report how often they performed each behavior, with scoring as never (0 points), rarely (1 point), sometimes (2 points), frequently (3 points).

Previous research has shown that touching and talking to the belly is widely recognized and measured as an indicator of maternal-fetal attachment [2, 22]. To better align with the literature, we created a binary outcome variable for analyses, that combined communication-related ESB performed by the expectant mother. These behaviors were touching/talking, singing, and telling about family. Dancing was not included because it does not necessarily involve directed sound or touch. Women who reported sometimes or frequently to any of the three communication behaviors were one category and women who reported only rarely or never were in the other.

Intimate partner communication was measured using the communication subscale of the Relationship Quality Index (RQI) [23]. The Constructive Communication subscale is divided into a three-question constructive and a four-question destructive scale. Responses were on a 5-point Likert scale, ranging from very unlikely [1] to very likely [5]. The four ‘destructive’ items were reverse coded and then all seven items were summed to create the total intimate partner communication score [24]. Constructive communication items included couples discuss problems, express feelings, and suggest solutions and compromises. Destructive communication items included couples blame each other, threaten with negative consequences, male partner calls the woman names and attacks her character, and female partner calls the man names and attacks his character.

Mental health was assessed using the Patient Health Questionnaire (PHQ-9), a common depression screener that has been validated for use among pregnant women in Ghana [25]. The PHQ-9 has nine items that are summed for a score between 0 and 27 and standard categorization: minimal or no depression (score of 0 to 4), mild depression (5–9), moderate depression (10 to 14), moderately severe depression (15–19), and severe depression (20–27) [26]. For analysis, we dichotomized the PHQ-9 into none to mild depression (score of less than 10) and moderate to extremely severe (score of 10 or greater), based on those who would have screened for no treatment or treatment in a clinical setting [25, 27]. Cronbach’s alpha for the PHQ-9 was 0.815, indicating high internal consistency.

Food insecurity was assessed using the Household Hunger Scale (HHS), a six-question scale with three main questions [went 24 h with no food, went to sleep hungry, and no food in the house due to lack of resources], each of which are followed by a question asking how often this event occurred. Questions are scored 0–6, with higher scores indicating higher food insecurity. For analysis, we used the standard categorical variables indicating little to no hunger (0–1 points), moderate to severe hunger (2–3 points), and severe hunger (4–6 points) [28].

Intimate partner violence (IPV) during the past 12 months was assessed using items from the 2008 Ghana Demographic and Health Survey (DHS). The four domains were controlling behaviors, physical IPV, sexual IPV, and emotional IPV [29]. Binary variables were created for each domain, indicating if the respondent endorsed at least one item within that domain.

Participants’ hopefulness was measured using the 12-item Herth Hope Index and analyzed as a continuous variable, with higher scores indicating higher hopefulness [30, 31]. The Cronbach’s alpha was 0.793, indicating high internal consistency.

Four questions were asked to determine participants’ perceived level of social support. These questions asked how much assistance you received in the past month from your husband/partner, female relatives, male friends, or female friends. Response options were sufficient, insufficient, or never received social support, and each question were recoded as a binary variable (never/insufficient support or sufficient support).

Additional questions included relationship status, woman’s age, parity, whether they had ever attended formal education, and self-reported physical health.

### Data management and analysis

Data collected via CommCare was uploaded and synced with the main database and analyzed using Stata version 16.1. A generalized estimating equations (GEE) modified Poisson model was used for bivariate and multivariable analyses, taking into account clustering by using an exchangeable working correlation [32]. The Kauermann-Carroll bias correction was used to account for potential small-sample bias in the standard errors, since the trial had fewer than 40 clusters [33, 34]. For the bivariate model, the correlation between the early stimulation factor score and each covariate was evaluated. The multivariable model was determined by including both a priori variables, education and parity, and excluding non-significant variables at the *p*-value level of 0.10.

## Results

The majority of respondents were between 16 and 34 years of age (82.6%), lived with their romantic partner (89.8%), and had had two or more pregnancies (78.1%) (Table 1). About half of the women had ever attended formal school (51.3%) and one-fourth endorsed moderate to severe household hunger (27.0%). Approximately half of the women reported very good or excellent physical health (48.4%). Women reported varying levels of perceived *sufficient* social support depending on the relationship: 45.8% from their husbands, 22.7% from male relatives, 36.7% from female relatives, and 17.4% from female friends. Twenty percent of women reported having moderate to severe depression symptoms. All forms of IPV were common; 79.1% reported that their partners exhibited controlling behaviors, 44.6% reported emotional IPV, 28.4% reported physical IPV, and 19.5% of women reported sexual IPV.

**Table 1:** Socio-demographic characteristics and bivariate analysis by combined early stimulation behaviors adjusted for clustering by community, Northern Ghana, *N*=374

Variable	Total ***N*** = 374	Frequently/Sometimes Combined Communication ESB ***N*** = 200	Never/Rarely Combined Communication ESB ***N*** = 174	Risk Ratio (95% CI)	***p***-value
**Mean (SD) Hope Score** (min, max)	**38.1 (3.8)** **(27, 48)**	38.3 (3.8) (31, 48)	37.9 (3.8) (27, 47)	1.01 (0.98, 1.04)	0.345
**Mean (SD) Relationship Communication** (min, max)	**26.9 (4.5)** **(9,35)**	26.9 (4.3) (13, 35)	26.9 (4.7) (9, 35)	1.01 (0.98, 1.03)	0.487
**Mean (SD) Age** (min, max)	**27.0 (6.8)** **(16, 50)**	26.6 (6.1) (16, 41)	27.4 (6.4) (17, 50)	0.99 (0.97, 1.01)	0.463
**Age**					0.498
≤ 24	**40.4%**	41.0%	39.7%	REF	
25–34	**41.2%**	43.0%	39.1%	1.07 (0.83, 1.38)	
≥ 35	**18.4%**	16.0%	31.3%	0.89 (0.62, 1.29)	
**Mean (SD) # of Pregnancies** (min, max)	**3 (1.9)** **(1, 9)**	3.3 (1.9) (1, 9)	3.4 (1.9) (1, 9)	0.99 (0.94, 1.04)	0.732
**Number of pregnancies**					0.755
1 pregnancy	**21.9%**	23.0%	20.7%	REF	
2–3 pregnancies	**34.0%**	35.5%	32.2%	0.97 (0.75, 1.26)	
4 or more pregnancies	**44.1%**	41.5%	47.1%	0.91 (0.71, 1.18)	
**Ever Attended Formal School**	**51.3%**	54.5%	47.7%	1.10 (0.87, 1.38)	0.418
**Hunger**					0.777
Little to None	**73.0%**	71.5%	74.7%	REF	
Moderate	**25.1%**	26.0%	24.1%	1.02 (0.79, 1.31)	
Severe	**1.9%**	2.5%	1.2%	1.24 (0.66, 2.32)	
**Living with Partner**	**89.8%**	89.0%	90.8%	0.97 (0.76, 1.24)	0.815
**Physical Health**					0.165
Fair/Poor	**15.5%**	16.5%	14.4%	REF	
Good	**36.1%**	40.5%	31.0%	1.06 (0.69, 1.61)	
Very Good/Excellent	**48.4%**	43.0%	54.6%	0.85 (0.57, 1.27)	
**Controlling Behavior** (12 months) *N* = 354	**79.1%**	82.1%	75.6%	1.19 (0.90, 1.57)	0.220
**Emotional IPV** (12 months) *N* = 359	**44.6%**	50.3%	37.9%	1.19 (1.02, 1.39)	0.031
**Physical IPV** (12 months) *N* = 359	**28.4%**	36.3%	19.3%	1.35 (1.06, 1.73)	0.016
**Sexual IPV** (12 Months) *N* = 358	**19.5%**	23.4%	15.1%	1.27 (1.01,1.58)	0.038
**Depression (PHQ-9)**					0.002
None to Mild	**80.2%**	74.5%	86.8%	REF	
Moderate to Severe	**19.8%**	25.5%	13.2%	1.33 (1.12,1.58)	
**Sufficient Social Support from-**					
Husband	**45.8%**	44.4%	47.4%	0.99 (0.76, 1.29)	0.955
Male Relatives	**22.7%**	18.5%	27.6%	0.82 (0.60, 1.11)	0.191
Female Relatives	**36.9%**	29.5%	45.4%	0.74 (0.53, 1.03)	0.074
Female Friends	**17.4%**	14.0%	21.3%	0.80 (0.59, 1.08)	0.137

The overall frequency of early stimulation behaviors performed by expectant mothers and other family members is shown in Table 2. A little over half (53.4%) of the women reported performing some type of communication-related behavior with their child in-utero, including touching and/or talking, singing, or telling about family to the belly (Table 2). While 25% of the women reported dancing with their belly sometimes or frequently, 15% said they encouraged older children to touch and/or talk to their belly and 25% reported encouraging their husbands/partners to touch and/or talk to their belly.

**Table 2:** Early Stimulation Behaviors Performed by Pregnant Women and their Families, Northern Ghana (*N* = 374)

Early Stimulation Behavior	Total n (%)
**Performed by pregnant women:**	
**Touching and/or talking to the belly**	
Never	158 (42.2)
Rarely	43 (11.5)
Sometimes	132 (35.3)
Frequently	41 (11.0)
**Singing**	
Never	267 (71.4)
Rarely	40 (10.7)
Sometimes	58 (15.5)
Frequently	9 (2.4)
**Telling about family**	
Never	284 (75.9)
Rarely	36 (9.6)
Sometimes	49 (13.1)
Frequently	5 (1.4)
**Dancing**	
Never	240 (64.2)
Rarely	39 (10.4)
Sometimes	89 (23.8)
Frequently	6 (1.6)
**Any communication combined (touch and/or talking, singing, and/or telling about family)**	
Never	136 (36.4)
Rarely	38 (10.2)
Sometimes	152 (40.6)
Frequently	48 (12.8)
**Performed by Family Members:**	
**Encourage older children to touch/ talk to the belly**	
Never	290 (77.5)
Rarely	28 (7.5)
Sometimes	48 (12.8)
Frequently	8 (2.1)
**Encourage partner/husband to touch/talk**	
Never	238 (63.6)
Rarely	44 (11.8)
Sometimes	79 (21.1)
Frequently	13 (3.5)

Bivariate analyses of participant characteristics by frequency of communication-related ESB are illustrated in Table 2. Results reveal that having moderate to severe depression symptoms was significantly associated with women performing communication-related ESB more frequently [Risk Ratio (RR) = 1.33, 95% Confidence Interval (CI) = (1.12, 1.58)]. Women were also more likely to communicate with their belly if they had experienced higher incidence of emotional [RR = 1.19, 95% CI = (1.02, 1.57)], physical [RR = 1.35, 95% CI = (1.06, 1.73)], and sexual [RR = 1.27, CI = (1.01, 1.58)] IPV. Increased social support from female relatives was negatively associated with performing communication-related ESB [RR = 0.74, 95% CI (0.53, 1.03)].

Emotional, physical, and sexual IPV were all highly correlated, so three separate multivariable models were run to include only one form of IPV at a time (Table 3). Parity, education, depression status, and female relative social support were included in each model. Maternal age was not included because it was highly correlated with parity. In the multivariable model, the effect of higher incidence of physical IPV [RR = 1.31, 95% CI = (1.01,1.69)] and sexual IPV [RR = 1.23, 95% CI = (0.97, 1.55)] on frequency of ESB was attenuated but remained relatively large.

**Table 3:** Multivariable model among pregnant women by early stimulation behavior adjusted for clustering by community, Northern Ghana, *N*=374

Predictor	Multivariable^**a**^ RR (95% CI)	***p***-value^**a**^	Multivariable^**b**^ RR (95% CI)	***p***-value^**b**^	Multivariable^**c**^ RR (95% CI)	***p***-value^**c**^
**Emotional IPV**	1.14 (0.95, 1.37)	0.142	–	–	–	–
**Physical IPV**	–	–	1.31 (1.01, 1.69)	0.039	–	–
**Sexual IPV**	–	–	–	–	1.23 (0.97, 1.55)	0.082
**# of Pregnancies**		0.511		0.536		
1 Pregnancy	REF		REF		REF	0.388
2–3 Pregnancies	0.94 (0.77, 1.16)		0.97 (0.79, 1.19)		0.93 (0.75, 1.15)	
4+ Pregnancies	0.88 (0.69, 1.11)		0.88 (0.69, 1.11)		0.86 (0.68, 1.07)	
**Ever Attended Formal School**	1.11 (0.90, 1.37)	0.323	1.09 (0.88, 1.36)	0.412	1.09 (0.88, 1.36)	0.417
**Depression (PHQ-9)**		0.097		0.148		0.085
None to Mild	REF		REF		REF	
Moderate to Severe	1.19 (0.97,1.46)		1.16 (0.95,1.41)		1.19 (0.97,1.45)	
**Sufficient Social Support from Female Relatives**	0.75 (0.50, 1.08)	0.140	0.75 (0.51, 1.10)	0.135	0.73 (0.49, 1.10)	0.128

^a^Model includes emotional IPV, but not physical or sexual IPV

^b^Model includes physical IPV, but not emotional or sexual IPV

^c^Model includes sexual IPV, but not emotional or physical IPV

## Discussion

This study helps illuminate the prevalence and correlates of early stimulation behaviors in a pregnant population in Northern Ghana. Data indicate that a little over half of women routinely performed communication-related ESB. There is sparse research on how frequently ESB during pregnancy should be performed or what specific behaviors lead to optimal developmental outcomes; however, some evidence suggests these behaviors may lead to higher maternal-fetal attachment [13]. Higher maternal-fetal attachment can lead to a stronger maternal-infant relationship and the maternal ability to care for and create a nurturing environment for her child, all of which can potentially lead to a reduced risk of developmental delays [1, 14]. The data indicate room for improvement when it comes to performing ESB during pregnancy; particularly if these early stimulating behaviors can help foster positive caregiver-child relationships and more responsive caregiving during infancy, which we know can improve developmental outcomes for children [35]. Likewise, the data indicate that other family members could be even more involved with the pregnancy (with the pregnant woman’s permission), thus laying the foundation for a wider familial responsive caregiving environment. Early childhood development programs during pregnancy that focus on building these prenatal relationships are scarce, but could have long-term affects when it comes to creating a healthy environment in which a child could thrive.

Our data showed that women who experienced more physical and/or sexual IPV were more likely to perform ESB during pregnancy. There could be several reasons for this response. One theory is that the pregnant woman is trying to protect her developing fetus from harm in cases of IPV, and therefore, touches and holds her belly as a way to reduce the fetus’ exposure to physical violence. In addition, we found that the frequency of ESB among those with moderate to severe depression symptoms was large and significant in the bivariate model but attenuated in the multivariable model. However, these results are still informative for future inquiry [1, 14]. One potential explanation for an increase in ESB when an expectant mother is faced with mental health difficulties, is that this action is used as a coping mechanism. The expectant mother might feel the most connected emotionally to her developing fetus and therefore turns to that maternal-fetal relationship for reassurance or self-soothing. This aligns with the attachment theory that states that attachment involves seeking care from someone who can provide security and comfort [36, 37]. This theory is usually used to describe the fetus’ relationship to their mother; however, in this case, the expectant mother is seeking comfort from her pregnant belly when she is faced with hardships in her life. One thing is clear—poor mental health, domestic violence, and household hunger are common amongst our study population and have been previously established as correlated with each other [18]. Any examination of ESB by pregnant women must be contextualized by the daily stressors of their lives.

There were both study limitations and strengths. First, as a cross-sectional study, we do not know the direction of the associations. We could not determine the timing of increases in ESB when faced with IPV or moderate to severe depression. Second, the study collected data in two districts in northern Ghana and is therefore not generalizable to the general population. However, study strengths include a robust sample size from across 32 communities in rural Northern Ghana with no previous data on ESB during pregnancy, and in fact, this is one of the only studies on correlates of ESB in the region.

## Conclusion

Little is known about the long-term impact of ESB during pregnancy on child development. However, we know that stimulation-focused interventions for caregivers are effective in creating more nurturing home environments, improving mother-child interactions, and increasing maternal knowledge [35]. Responsive caregiving is a continuum and further research is needed to better understand its role throughout different stages of early childhood development.

## Supplementary Information

**Additional file 1.** Evaluation of the iMBC/ECD Model on Maternal Mental Health and Child Development in Ghana Baseline survey.

## Data Availability

The dataset generated and/or analyzed during the current study is not publicly available but is available via communication with the corresponding author (JM) and the senior author (JNB) on reasonable request, with approval from CRS, and with IRB approval to maintain confidentiality.

## Abbreviations

ESB: Early stimulation behaviors
ECD: Early childhood development
IPV: Intimate partner violence
RA: Research assistants
DHS: Demographic and Health Survey
GEE: Generalized estimating equations
PHQ-9: Patient Health Questionnaire-9 item version
HHS: Household hunger scale
iMBC: Integrated Mothers and Babies Course
RQI: Relationship quality index
CRS: Catholic Relief Services
WHO: World Health Organization
REST II: Rural Emergency Health Service and Transport
C-PrES: Community Surveillance and Targeted Education Sessions
