# Supplementary material for: Prevalence and correlates of maternal early stimulation behaviors during pregnancy in northern Ghana: a cross-sectional survey
Source: BMC Pregnancy Childbirth. 2021 Jan 4;21:4. doi: 10.1186/s12884-020-03476-9 (PMC7784360; doi:10.1186/s12884-020-03476-9)
Supplement: Supplementary file 1 — Additional file 1. Evaluation of the iMBC/ECD Model on Maternal Mental Health and Child Development in Ghana Baseline survey. [file 12884_2020_3476_MOESM1_ESM.pdf]

Evaluation of the iMBC/ECD Model on Maternal Mental Health and Child Development in Ghana

Baseline survey

SECTION 0: Consent Review

Introduction:

Hello, my name is ________________. I am working with Catholic Relief Services to conduct research on the topic of maternal health and childhood development.

[Review consent with interviewee.]

Before beginning the research we want to remind you:

• Your participation is entirely voluntary;

• You may decide not to take part or to withdraw from the study at any time

• Your decision to participate or withdraw from the study will not affect your participation in the C-PrES groups.

The purpose of this study is to understand how participation in the mothers group will affect your mental health and the development of your child.

You will be interviewed four times over the next 2 years. Your participation in the study will end at the end of the fourth interview.

Thank you for your willingness to participate in our study. Today is our first interview out of four interviews. The interview will take about 1 hour to complete. As I just described, I will ask you a number of questions about you and your baby during the interview today. During each interview after this one, we'll talk through similar questions.

Do you have any questions before we begin?

SECTION 1: Contact information

**S1Qa_0**	What is your home address?<br>*[Enter address and repeat back to respondent to confirm if correct.]*<br>________________________________	
**S1Qb_0**	Can those who are collecting data come to your home to contact you?<br>*00. No 01. Yes*	|___||___|
**S1Qc_0**	Do you have a functioning mobile phone?<br>*00. No 01. Yes*<br>*If "yes" skip to* **S1Q01_0**	|___||___|
**S1Q01_0**	What is the best phone number we can use to call you (to schedule appointment, to send a reminder about an appointment)?<br>*[Enter number and repeat back to respondent to confirm if correct.]*<br>________________________________	
**S1Q02_0**	May we call this number?<br>*00. No 01. Yes*	|___||___|
**S1Q05_0**	When I call, may I mention that you are part of the mothers groups?<br>*00. No 01. Yes*	|___||___|
**S1Q06_0**	Do you have a second phone number we can use to call you (to schedule appointment, to send a reminder about an appointment)?<br>*[Enter number and repeat back to respondent to confirm if correct.]*	

	____________

**S1Q07_0**	May we call this number? *00. No 01. Yes*	|___||___|
**S1Q10_0**	When I call may I mention that you are part of the C-PreS/Mothers group? *00. No 01. Yes*	|___||___|

SECTION 2: Reproductive and health history

Next I would like to better understand your health history.

[Interviewer fills out grid below, with mother's answers to these questions]

**S2Q01_0**	How many times have you become pregnant (live births/miscarriages/stillbirths)? Interviewer: If the woman is pregnant, please ask her to include current pregnancy in the answer.<br><br>*[For 2, enter 02, etc…]*	|___||___| If 1 (i.e., first pregnancy, skip to section 3)

Pregnancy #	S2Q02_0<br>**What was the outcome of each pregnancy? Skip if answer to previous question is 1**<br>*01 = Born alive*<br>*02 = Not Born alive**<br>**miscarriage or still birth*	S2Q03_0<br>**Age of child**<br>*77. If born alive and has since died*<br>*Years*	*Months*	S2Q04_0<br>**Breastfeeding now?**<br>*[If living]*<br>*00 = No*<br>*01 = Yes*	S2Q05_0<br>*Any comments about pregnancies? (e.g. di the mother have twin*
1st	|___||___|	|___||___|	|___||___|	|___||___|	
2nd	|___||___|	|___||___|	|___||___|	|___||___|	
3rd	|___||___|	|___||___|	|___||___|	|___||___|	
4th	|___||___|	|___||___|	|___||___|	|___||___|	
5th	|___||___|	|___||___|	|___||___|	|___||___|	
6th	|___||___|	|___||___|	|___||___|	|___||___|	
7th	|___||___|	|___||___|	|___||___|	|___||___|	
8th	|___||___|	|___||___|	|___||___|	|___||___|	
9th	|___||___|	|___||___|	|___||___|	|___||___|	
10th	|___||___|	|___||___|	|___||___|	|___||___|	

EARLY STIMULATION<br>*Questions adapted from examples of early stimulation behaviors used in the iMBC program.*						
During your current pregnancy, how often do you do the following?<br>00 = Never; 01= Rarely; 02=Sometimes; 03=Frequently						
	**S2Q16_0**<br>Talk softly to him/her & touch belly?	**S2Q17_0**<br>Sing songs to him/her?	**S2Q18_0**<br>Tell him/her about his/her family?	**S2Q19_0**<br>Dance to music or the radio?	**S2Q20_0**<br>Encourage older children to touch & talk? (in family or community)	**S2Q21_0**<br>Encourage partner/husband to talk & touch?
	|___||___|	|___||___|	|___||___|	|___||___|	|___||___|	|___||___|

Health Survey		
**S2Q22_0**	In general, would you say your health is:<br>*[READ OPTIONS ALOUD]*<br>01 = Excellent<br>02 = Very Good<br>03 = Good<br>04 = Fair<br>05 = Poor	|___||___|
**S2Qa_0**	Do you have a chronic illness?<br>01=Yes<br>00=No→ If no skip to next section	|___||___|
**S2Qb_0**	Which illness or illnesses? (choose all that apply)<br>01- Tuberculosis<br>02- Diabetes<br>03- Hypertension<br>04- HIV/AIDS<br>05- Other (SPECIFY_____________________________________)	|___||___| |___||___| |___||___| |___||___|

SECTION 6: Social Support

Thank you. The next questions ask for your views about your health, such as how you have been feeling and how well you are able to do your usual activities.

For each of the following questions, please select the response that best describes how you have been feeling. If you are unsure about how to answer a question, please give the best answer you can.

**Social Support**		
**S6Q01_0**	In the last month, how much assistance did you receive from your **husband/partner**<br>01 = Sufficient: I received the attention and assistance that I needed<br>00 = Insufficient: I would have liked more attention and assistance<br>03=Never<br>04= Do not have husband/partner.	|___||___|
**S6Q02_0**	In the last month, how much assistance did you receive from your **female relatives** (either side of the family).<br>01 = Sufficient: I received the attention and assistance that I needed<br>00 = Insufficient: I would have liked more attention and assistance<br>03=Never	|___||___|
**S6Q03_0**	In the last month, how much assistance did you receive from your **male relatives** (either side of the family).<br>01 = Sufficient: I received the attention and assistance that I needed<br>00 = Insufficient: I would have liked more attention and assistance<br>03=Never	|___||___|
**S6Q04_0**	In the last month, how much assistance did you receive from your **female friends**<br>01 = Sufficient: I received the attention and assistance that I needed<br>00 = Insufficient: I would have liked more attention and assistance<br>03=Never	|___||___|
**Modified Medical Outcomes Study Social Support (mMOS-SS) Survey**<br><br>*Moser, A., Stuck, A. E., Silliman, R. A., Ganz, P. A., & Clough-Gorr, K. M. (2012). The eight-item modified Medical Outcomes Study Social Support Survey: psychometric evaluation showed excellent performance. Journal of clinical epidemiology, 65(10), 1107–1116. https://doi.org/10.1016/j.jclinepi.2012.04.007*		
People sometimes look to others for companionship, assistance, or other types of support. How often is each of the following kinds of support available to you if you need it?<br>Response categories:<br>00=None of the time<br>01=A little of the time<br>02=Some of the time<br>03=Most of the time<br>04=All of the time		
**S6Q04_0**	If you needed it, how often is someone available to help you if you were confined to bed?	|___||___|
**S6Q05_0**	…to take you to the doctor if you need it?	|___||___|

**S6Q06_0**	…to prepare your meals if you are unable to do it yourself?	|___||___|
**S6Q07_0**	…to help with daily chores if you were sick?	|___||___|
**S6Q08_0**	…to have a good time with?	|___||___|
**S6Q09_0**	…to turn to for suggestions about how to deal with a personal problem?	|___||___|
**S6Q10_0**	…who understands your problems?	|___||___|
**S6Q11_0**	…to love and make you feel wanted?	|___||___|

SECTION 7: Household hunger

**Household Hunger Scale**<br>*Ballard T, Coates J, Swindale A, Deitchler M (2011) Household Hunger Scale: Indicator Definition and Measurement Guide. Food and Nutrition Technical Assistance II Project, FHI 360, Washington, DC*		
**S7Q01_0**	In the past [4 weeks/30 days], did you or any household member go a whole day and night without eating anything at all because there was not enough food?<br>00 = No → **SKIP TO S7Q05_0**<br>01 = Yes<br>88/99 = DK/Refused → **SKIP TO S7Q05_0**	|___||___|
**S7Q02_0**	How often did this happen in the past [4 weeks/30 days]?<br>01 = Rarely (1-2 times)<br>02 = Sometimes (3-10 times)<br>03 = Often (More than 10 times)	|___||___|
**S7Q03_0**	In the past [4 weeks/30 days], did you or any household member go to sleep at night hungry because there was not enough food?<br>00 = No → **SKIP TO S7Q03_0**<br>01 = Yes<br>88/99 = DK/Refused → **SKIP TO S7Q03_0**	|___||___|
**S7Q04_0**	How often did this happen in the past [4 weeks/30 days]?<br>01 = Rarely (1-2 times)<br>02 = Sometimes (3-10 times)<br>03 = Often (More than 10 times)	|___||___|
**S7Q05_0**	In the past 4 weeks (30 days), was there ever no food to eat of any kind in your house because of lack of resources to get food?<br>00 = No → **SKIP TO S7Q07_0**<br>01 = Yes<br>88/99 = DK/Refused → **SKIP TO S7Q07_0**	|___||___|
**S7Q06_0**	How often did this happen in the past [4 weeks/30 days]?	

	01 = Rarely (1-2 times)<br>02 = Sometimes (3-10 times)<br>03 = Often (More than 10 times)	|___||___|
**S7Q07_0**	In the past 4 weeks (30 days), did you worry that your household would not have enough food?<br>00 = No → **SKIP TO Section 8**<br>01 = Yes<br>88/99 = DK/Refused → **SKIP TO Section 8**	|___||___|
**S7Q08_0**	How often did this happen in the past [4 weeks/30 days]?<br>01 = Rarely (1-2 times)<br>02 = Sometimes (3-10 times)<br>03 = Often (More than 10 times)	|___||___|

SECTION 8: Mental Health

**Self-Reporting Questionnaire (SRQ-20)**<br>*Beusenberg, M., Orley, J. H., & World Health Organization. (1994). A User's guide to the self reporting questionnaire (SRQ (No. WHO/MNH/PSF/94.8. Unpublished). World Health Organization.*		
**Now I'd like you to think about the past 30 days and how you have felt during that time.**<br>00 = No<br>01 = Yes		
**S8Q01_0**	Do you often have headaches? *(Note: past 30 days)*	|___||___|
**S8Q02_0**	Is your appetite poor? (*Note: past 30 days)*	|___||___|
**S8Q03_0**	Do you sleep badly? *(Note: past 30 days)*	|___||___|
**S8Q04_0**	Are you easily frightened? *(Note: past 30 days)*	|___||___|
**S8Q05_0**	Do your hands shake? *(Note: past 30 days)*	|___||___|
**S8Q06_0**	Do you feel nervous, tense or worried? *(Note: past 30 days)*	|___||___|
**S8Q07_0**	Is your digestion poor? (*Note: past 30 days)*	|___||___|
**S8Q08_0**	Do you have trouble thinking clearly? (*Note: past 30 days)*	|___||___|
**S8Q09_0**	Do you feel unhappy? *(Note: past 30 days)*	|___||___|

**S8Q10_0**	Do you cry more than usual? (*Note: past 30 days)*	|___||___|
**S8Q11_0**	Do you find it difficult to enjoy your daily activities? *(Note: past 30 days)*	|___||___|
**S8Q12_0**	Do you find it difficult to make a decision? *(Note: past 30 days)*	|___||___|
**S8Q13_0**	Is your daily work suffering? *(Note: past 30 days)*	|___||___|
**S8Q14_0**	Are you unable to play a useful part in life? *(Note: past 30 days)*	|___||___|
**S8Q15_0**	Have you lost interest in things? *(Note: past 30 days)*	|___||___|
**S8Q16_0**	Do you feel that you are a worthless person? *Note: past 30 days)*	|___||___|
**S8Q17_0**	Has the thought of ending your life been on your mind? *(Note: past 30 days. A positive response requires interviewer to provide referral after interview.)*	|___||___|
**S8Q18_0**	Do you feel tired all the time? *(Note: past 30 days)*	|___||___|
**S8Q19_0**	Do you have uncomfortable feelings in your stomach? *(Note: past 30 days)*	|___||___|
**S8Q20_0**	Are you easily tired? *Note: past 30 days)*	|___||___|

Patient Health Questionnaire (PHQ-9)

Kroenke K, Spitzer RL. The PHQ-9: A new depression diagnostic and severity measure. Psychiatr Ann Thorofare. 2002 Sep;32(9):509–15.

Over the last 2 weeks, how often have you been bothered by any of the following problems?

00-Not at all
01-Several Days
02- More than half the days
03- Nearly everyday

**S8Q21_0**	Little interest or pleasure in doing things	|___||___|
**S8Q22_0**	Feeling down, depressed, or hopeless	|___||___|
**S8Q23_0**	Trouble falling or staying asleep, or sleeping too much	|___||___|
**S8Q24_0**	Feeling tired or having little energy	|___||___|
**S8Q25_0**	Poor appetite or overeating	|___||___|
**S8Q26_0**	Feeling bad about yourself — or that you are a failure or have let yourself or your family down	|___||___|

**S8Q27_0**	Trouble concentrating on things, such as reading the newspaper or watching television	|___||___|
**S8Q28_0**	Moving or speaking so slowly that other people could have noticed? Or the opposite — being so fidgety or restless that you have been moving around a lot more than usual	|___||___|
**S8Q29_0**	Thoughts that you would be better off dead or of hurting yourself in some way (*A positive response requires interviewer to provide referral after interview.)*	|___||___|
**S8Q30_0**	If you checked off any problems, how difficult have these problems made it for you to do your work, take care of things at home, or get along with other people?<br><br>01- Not difficult at all<br>02- Somewhat difficult<br>03- Very Difficult<br>04- Extremely Difficult	|___||___|

SECTION 9: Couple Functionality and Intimate Partner Violence (IPV)

**Relationship status**		
**S9Q01_0**	*What is your current relationship status?*<br>01 = Married and living with your husband<br>02 = Married and not living with your husband<br>03 = Living with a romantic partner whom you are not married to→ skip to **S9QA_0**<br>04 = With romantic partner but not married to nor living together skip to **S9QA_0**<br>05= No current partner → skip to **S9Q01c_0**	|___||___|
**S2Q01b_0**	*Are you married to a man who has more than one wife?*<br>01=yes<br>00= no<br><br>➔ Skip to **S9QA_0**	|___||___|
**S9Q01c_0**	*Are you currently…*<br>01-widowed<br>02-divorced<br>03-separated<br>04- Single, never married<br><br>➔ Skip to S9Q02b	|___||___|
**S9QA_0**	*Does your husband or partner attend any health related education groups?*<br>01=yes<br>00= no<br>88= don't know	|___||___|

S9Q02_0	*Did you have a husband or partner in the last 12 months?*<br>00 – Yes<br>01 – No – section 10	|___||___|

Now I would like to ask you some questions about the relationship you have with your husband/partner.

**Communication subscale of the Couple Functionality Assessment**<br>*Ruark A, Chase R, Hembling J, Davis VR, Perrin PC, Brewster-Lee D. Measuring couple relationship quality in a rural African population: Validation of a Couple Functionality Assessment Tool in Malawi. PloS One. 2017;12(11).*						
Please tell me what you do when a problem arises in your relationship.<br>**skip if S9Q01a=** 05= No current partner						
		Very unlikely 01	Unlikely 02	Somewhat likely 03	Likely 04	Very likely 05
**S9Q03_0**	We try to discuss the problem					
**S9Q04_0**	We express our feelings to each other					
**S9Q05_0**	We suggest possible solutions and compromises					
**S9Q06_0**	We blame, accuse, and criticize each other. (R)					
**S9Q07_0**	We threaten each other with negative consequences. (R)					
**S9Q08_0**	I call my husband/partner names, swear at him, or attack his character. (R)					
**S9Q09_0**	My husband/partner calls me names, swears at me, or attacks my character. (R)					

**Intimate Partner Violence (IPV)**<br>*From the 2014 Ghana Demographic and Health Survey*	
	Check for presence of others: Do not continue until effective privacy is ensured.

	**Now I would like to ask you questions about some other important aspects of a woman's life. I know that some of these questions are very personal. Your answers are helpful in understanding your experiences. If you are uncomfortable or prefer not to answer, you do not have to answer these questions. Let me assure you that your answers are completely confidential and will not be told to anyone without your permission.**<br>*[These questions concern all women regardless of their relationship status, unless they have not had a partner in the last 12 months]*

	**When two people marry or live together, they share both good and bad moments. In your relationship with your husband/partner in the last 12 months did the following happen frequently, only sometimes, or never?**<br>01 = Frequently<br>02 = Sometimes<br>03 = Never	
**S9Q11_0**	He usually spends/spent his free time with you?	|___||___|
**S9Q12_0**	He (consults/consulted) you on different household matters?	|___||___|
**S9Q13_0**	He (is/was) affectionate with you?	|___||___|
**S9Q14_0**	He (respects/respected) you and your wishes?	|___||___|
	**Now I am going to ask you about some situations which happen to some women. Please tell me if these apply to your relationship with your husband/partner in the last 12 months**?<br>00 = No<br>01 = Yes<br>88 = Don't Know	
**S9Q15_0**	He (is/was) jealous or angry if you (talk/talked) to other men?	|___||___|
**S9Q16_0**	He frequently (accuses/accused) you of being unfaithful?	|___||___|
**S9Q17_0**	He (does/did) not permit you to meet your girl friends	|___||___|
**S9Q18_0**	He (tries/tried) to limit your contact with your family	|___||___|
**S9Q19_0**	He (insists/insisted) on knowing where you (are/were) at all time?	|___||___|
**S9Q20_0**	He (does/did) not trust you with any money?	|___||___|
	**Now if you will permit me, I need to ask some more questions about your relationship with your (last) husband/partner. In the last 12 months, has your husband/partner:**<br>00 = No<br>01 = Yes<br>88 = Don't Know	
**S9Q21_0**	Said or done something to humiliate you in front of others?	|___||___|
**S9Q22_0**	Threatened you or someone close to you with harm?	|___||___|

**S9Q23_0**	Insulted or belittled you?	|___||___|
**S9Q24_0**	Pushed you, shaken you, or thrown something at you? *(Note: positive responses to S9Q25-S9Q33 require interviewer to provide referral information after interview.)*	|___||___|
**S9Q25_0**	Slapped you?	|___||___|
**S9Q26_0**	Twisted your arm, or pulled your hair?	|___||___|
**S9Q27_0**	Punched you with his fist or with something that could hurt you ?	|___||___|
**S9Q28_0**	Kicked or dragged you?	|___||___|
**S9Q29_0**	Tried to strangle you or burn you ?	|___||___|
**S9Q30_0**	Threatened you with a knife, a gun, or other type of weapon ?	|___||___|
**S9Q31_0**	Attacked you with a knife, a gun, or other type of weapon ?	|___||___|
**S9Q32_0**	Physically forced you to have sexual intercourse with him even when you did not want to?	|___||___|
**S9Q33_0**	Forced you to perform other sexual acts you did not want to?	|___||___|

SECTION 10: Demographics and household composition

Demographic and Household Questions<br>*From the 2014 Ghana Demographic and Health Survey*		
**S10Q02_0**	How old were you at your last birthday?<br>ENTER AGE IN COMPLETED YEARS	|___||___|
**S10Q03_0**	Have you ever attended school?<br>00-No →Skip S10Q34 and S10Q35<br>01 -Yes	|___||___|
**S10Q04_0**	What is the highest level of school you attended: primary,vocational, secondary, or higher?<br>PRIMARY . . . . 1<br>POST-PRIMARY/VOCATIONAL . . . . . 2<br>SECONDARY/ 'A' LEVEL . . . . 3<br>COLLEGE (MIDDLE LEVEL) . . . . 4<br>UNIVERSITY 5	|___||___|
**S10Q05_0**	What is the highest (standard/form/year) you completed at that level?<br>IF COMPLETED LESS THAN ONE YEAR AT THAT LEVEL, RECORD '00'.	|___||___|
**S10Q07_0**	Including you, how many adults and children live in your household?	|___||___|
**S10Q08_0**	Of those in your household, how many are older than 18 years of age?	|___||___|
**S10Q09_0**	How many of those older than 18 in your household are female?	|___||___|
**S10Q10_0**	Male?<br>*[Check that the total of males and females over 18 matches earlier total. If not, ask for clarification.]*	|___||___|

**S10Q11_0**	Of those in your household, how many are under the age of 18?	|___||___|
**S10Q12_0**	How many of those under 18 in your household are female?	|___||___|
**S10Q13_0**	Male?<br>*[Check that the total of males and females under 18 matches earlier total. If not, ask for clarification.]*	|___||___|
**S10Q14_0**	How many children 5 years or less live in your home ?	|___||___|
**S10Q15_0**	How many children ages 6 – 18 years old attend school?	|___||___|
**S10QA_0**	Are you a part of a SILC group?<br>00=No<br>01=yes	
**S10Q16_0**	As you know, some people take up jobs for which they are paid in cash or kind. Others sell things, have a small business or work on the family farm or in the family business.<br>Have you done any work in the last 7 days<br>00=No<br>01=yes → Skip to S10Q18_0 (occupation)	|___||___|
**S10Q17_0**	Have you done any work in the last 12 months?<br>00=No → Skip to S10Q20_0<br>01=yes	|___||___|
**S10Q18_0**	What is your occupation, that is, what kind of work do you mainly do?<br>01-professional/technical/managerial<br>02- clerical<br>03-sales and services<br>04-Skilled Manual<br>05- Unskilled manual<br>06-Agriculture<br>07-Not currently working	|___||___|
**S10Q19_0**	Are you paid in cash or kind for this work or are you not paid at all?<br>03-paid in cash or kind	|___||___|

	02-paid in cash<br>01-paid in kind<br>00-not paid at all	
**2016 Ghana Equity Tool**<br><br>*https://www.equitytool.org/ghana-old-page/*		
**S10Q21_0**	Does your household have a: color television?<br>00-No<br>01- Yes	|___||___|
**S10Q22_0**	Does any member of this household own any agricultural land?<br>00-No<br>01- Yes	|___||___|
**S10Q23_0**	Refrigerator?<br>00-No<br>01- Yes	|___||___|
**S10Q24_0**	Video deck/DVD/VCD<br>00-No<br>01- Yes	|___||___|
**S10Q25_0**	Does any member of this household have a bank account (not mobile money)?<br>00-No<br>01- Yes	|___||___|
**S10Q26_0**	Electricity?<br>00-No<br>01- Yes	|___||___|
**S10Q27_0**	A clock (access to time)?<br>00-No<br>01- Yes	|___||___|

**S10Q28_0**	Cabinet/cupboard?<br>00-No<br>01- Yes	|___||___|
**S10Q29_0**	What type of fuel does your household mainly use for cooking?<br>00- LPG<br>01 – Wood<br>03- Charcol<br>02- Other	|___||___|
**S10Q30_0**	What kind of toilet facility do members of your household usually use?<br>00- No facility/bush/field<br>01-Other	|___||___|
**S10Q31_0**	What is the main source of drinking water for members of your household?<br>00- Sachet water<br>01-Other	|___||___|
**S10Q32_0**	What is the main material of the floor in your household?<br>00-Cement<br>01- Other	|___||___|
**S10Q33_0**	What is the main material of the exterior walls in your household?<br>00 – Cement<br>01- Other	|___||___|

**S10Q34_0**	Do you use or anyone in your household use mobile money?<br>(added by Ghana team)<br>00-No<br>01- Yes	|___||___|
**S10Q35.**	Does your household use a satellite dish?<br>(added by Ghana team)<br>00-No<br>01- Yes	|___||___|

Section 11: Hope

Listed below are a number of statements. As I read each statement to you, please think about how much you agree with that statement right now.

**Herth Hope Index**<br>*Herth K. Abbreviated instrument to measure hope: development and psychometric evaluation. J Adv Nurs. 1992;17(10):1251–9.*					
		**STRONGLY DISAGREE**	**DISAGREE**	**AGREE**	**STRONGLY AGREE**
**S11Q01_0**	I have a positive outlook toward life.	00	01	02	03
**S11Q02_0**	I have short and/or long range goals.	00	01	02	03
**S11Q03_0**	I feel all alone	03	02	01	00
**S11Q04_0**	I can see possibilities in the midst of difficulties.	00	01	02	03
**S11Q05_0**	I have a faith that gives me comfort.	00	01	02	03
**S11Q06_0**	I feel scared about my future.	03	02	01	00
**S11Q07_0**	I can recall happy/joyful times.	00	01	02	03
**S11Q08_0**	I have deep inner strength.	00	01	02	03
**S11Q09_0**	I am able to give and receive caring/love.	00	01	02	03
**S11Q10_0**	I have a sense of direction.	00	01	02	03
**S11Q11_0**	I believe that each day has potential.	00	01	02	03
**S11Q12_0**	I feel my life has value and worth.	00	01	02	03

Section 12: Health Service Utilization

Self Reported- Antenatal Care		
S12Q01_0	Did you see anyone for antenatal care for this pregnancy?<br><br>01- Yes<br>02- 00-No -→ Skip to S12Q04_0<br>03- 99 Do not know/No Response → Skip to S12Q04_0	|___||___|
S12Q02_0	Whom did you see?<br>01- Doctors<br>02- Nurse/Midwife/Community Health Officer<br>03- Traditional Birth Attendant<br>04- Community Health Volunteer<br>05- Traditional Health Practitioner<br>06- Other (Specify)_______________________________)	|___||___|
S12Q03_0	Where did you receive antenatal care for this pregnancy?<br>01- Your home<br>02- Hospital<br>03- Health Center<br>04- Government Health Post (CHPS)<br>05- Mobile Clinic<br>06- Other (specify _____________________________)	|___||___|
S12Q04_0	How many months pregnant were you when you first received antenatal care?	|___||___|
S12Q05_0	How many times did you receive antenatal care during this pregnancy?<br><br>**IF DONT KNOW ENTER « 99 »**	|___||___|

Section 13: Referral

**S13Q01_0**	**Interviewer**: Was a referral for mental health offered/provided to the participant? *[Note: Refer to questions S9Q25-S9Q33]*<br>*00. No 01. Yes*	|___||___|
**S13Q02_0**	**Interviewer**: Was a referral for intimate partner violence offered/provided to the participant? *[Note: Refer to questions S9Q25-S9Q33]*<br>*00. No 01. Yes*	|___||___|

Read :

End Option 1 : No Referrals

End Option 2 : Referral for IPV or Mental Health

SECTION 14: Checklist

**S14Q01_0**	Reviewed consent (remind, review original) or administered consent to new participant?<br>*00. No*<br>*01. Reviewed consent with original participant*<br>*02. Administered informed consent with new participant*	|___||___|
**S14Q02_0**	Asked participant if they had any questions<br>*0. No 1. Yes*	|___|
**S14Q03_0**	Contact section updated<br>*0. No 1. Yes*	|___|
**S14Q04_0**	Survey completed<br>*0. No 1. Yes*	|___|
**S14Q05_0**	Participant ID is written at the top of each page<br>0. No 1. Yes	|___|
**S14Q06_0**	Date is written at the top of each page<br>0. No 1. Yes	|___|
**S14Q07_0**	Double-check that all information has been completed before ending the visit<br>*0. No 1. Yes*	|___|
**S14Q08_0**	Remind mother/caregiver of next visit date (*~7 months)*<br>*0. No 1. Yes*	|___|
**S14Q09_0**	Incentive was given out and incentive paperwork was completed<br>*0. No 1. Yes*	|___|
**S14Q11_0**	Place of interview<br>01 = *Health facility/clinic*<br>02 = *Participant's home*<br>03 = *Other, specify :* ______________________________	|___||___|
**S14Q12_0**	Interview site<br>01 = *West Mamprusi Municipality*<br>02 = *Nabdam District*<br>03 = *Other, specify :* ______________________________	
**S14Q13_0**	Notes (free text)	
**S14Q14_0**	Interviewer initials	
